# Survival Trend of Tuberculosis Patients and Risk Factors Associated with Mortality and Developing Drug-Resistant Tuberculosis in Hospital Pulau Pinang, Malaysia: A Retrospective Study

**DOI:** 10.3390/arm90060054

**Published:** 2022-11-14

**Authors:** Aseel Rezeq Yaghi, Heba Saleh Shaheed, Sabariah Noor Harun, Irfhan Ali Hyder Ali, Amer Hayat Khan

**Affiliations:** 1 Discipline of Clinical Pharmacy, School of Pharmaceutical Sciences, Universiti Sains Malaysia, Gelugor, Penang 11800, Malaysia; 2 Department of Medical Laboratory Techniques, Al-Mustaqbal University College, Hillah 51001, Iraq; 3 Respiratory Department, Penang General Hospital, Penang 10990, Malaysia

**Keywords:** multidrug-resistant, mortality, death, treatment outcomes, survival, unsuccessful treatment

## Abstract

**Highlights:**

**What are the main findings?**

- Higher rate of mortality among drug addict TB patients and an association with abnormal laboratory values.
- Relapsed TB and alcohol consumers are a high-risk population for developing MDR-TB.

**What is the implication of the main finding?**

- Assist clinicians in identifying high-risk patients prior to or early in the course of TB treatment, which helps improve treatment outcomes.
- Further research is necessary to identify risk factors associated with MDR-TB.

**Abstract:**

Background: Multidrug resistance TB (MDR-TB) has emerged as a public health issue worldwide, and the mortality rate is worrying. Therefore, this study was conducted to investigate the factors related to MDR-TB occurrence and the survival experience of TB patients. Methods: A retrospective cohort study was conducted at Hospital Pulau Pinang in Malaysia. Medical records of active TB patients from 2014–2018 were reviewed. Cox regression was used to identify the factors associated with MDR-TB development and mortality among TB patients. Results: The patients had a mean age of 48.84 ± 16.713 years, and a majority of the Chinese race (46.4%). Out of 351 TB patients, 325 (92.6%) were drug-susceptible TB, and 26 (7.4%) were diagnosed with MDR-TB. Among drug-susceptible TB patients, 245 (75.4%) achieved successful outcomes, and 73 (22.5%) passed away. In multivariable Cox regression, drug addiction, levels of white blood cells, urea, platelets, and albumin were significantly associated with death. Relapsed TB, alcohol consumption, and being single were significant risk factors for MDR-TB development. Conclusion: Patients achieved a success rate of 75.4%, which is encouraging but still far below the WHO target (at least an 85% success rate) and has room for further improvement.

## 1. Introduction

Tuberculosis (TB) is still a disease that has a strong impact on global public health. Efforts are made to control the disease transmission, especially in high TB burden, and to face the emerged multidrug-resistant TB (MDR-TB), which plays a key role in achieving unsuccessful TB treatment outcomes. Globally, TB is the leading infectious disease killer and one of the top ten causes of death, with 10.0 million people falling ill with TB in 2019 [1,2]. The impact of COVID-19 has resulted in a large drop in TB incidence due to the increase in mortality among TB patients, from 1.2 million in 2019 to 1.4 million in 2020 [2]. In Malaysia, an intermediate–TB burden country, the TB mortality rate is the highest death rate in comparison with other infectious diseases, showing that transmission of TB is still active [3]. It increased from 5.5/per 100,000 people in 2014 to 7.1 per 100,000 people in 2020 [4].MDR-TB, which is defined as the resistance of *Mycobacterium tuberculosis* to at least isoniazid and rifampicin, the most potent first-line anti-tubercular drugs, has appeared to compromise patients’ health and to challenge national TB control programs. A total estimate of 465,000 new MDR-TB cases were reported worldwide in 2019 [1]. Even though MDR-TB is relatively uncommon in Malaysia, the number of MDR-TB cases climbed from 101 to 370 between 2015 and 2017, which highlighted the need for further research into management and treatment outcomes [5]. The previous literature shows a scarcity of data on MDR-TB outcomes and related risk factors in Malaysia, especially after 2015. Moreover, this is the first study in the country to evaluate the effect of laboratory values (WBC, platelets, neutrophils, lymphocytes, ALT, albumin, urea, and creatinine) on TB-related mortality. The current study aimed to determine the factors that contribute to the development of MDR-TB, as well as the associated factors of mortality among TB patients.

## 2. Materials and Methods

### 2.1. Study Design and Population

**Figure 1:**
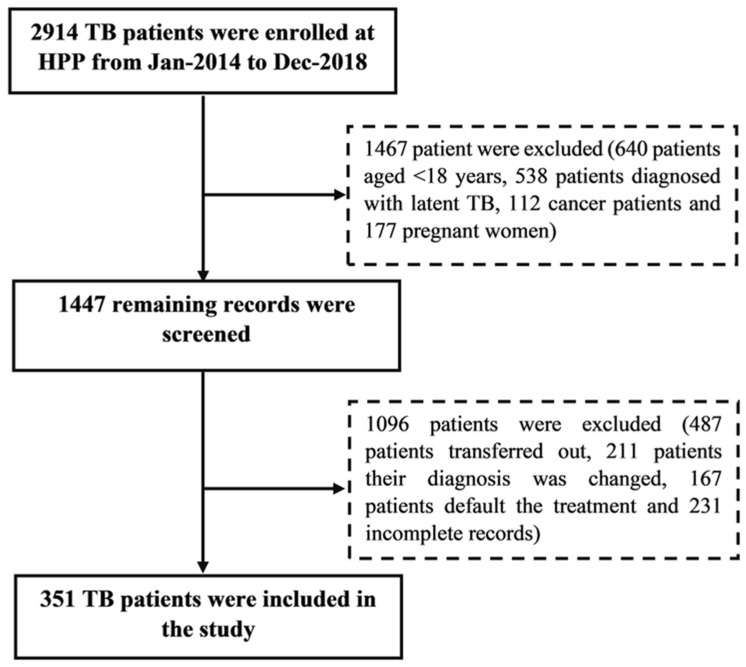
Enrollment, inclusion, and exclusion of study patients.

A retrospective cohort study was conducted at the respiratory clinic of Hospital Pulau Pinang (HPP), a public sector tertiary care referral hospital that covers a large proportion of the Pinang population (1.7 million), the 3rd populated state in Malaysia [6]. It is currently the second-largest tertiary health care public hospital with a capacity of 1107 beds in 1961 in the northern region of Malaysia. Medical records of patients with confirmed TB diagnoses from 2014–2018 were screened, and a total of 351 patients were included in the final analysis. Patients aged 18 years or above, diagnosed with active pulmonary tuberculosis (PTB) and/or extrapulmonary tuberculosis (EPTB), patients diagnosed with MDR-TB, and patients with co-morbidities with TB, including diabetes mellitus, hepatitis B virus, hepatitis C virus, and human immunodeficiency virus, were included in the study, whereas patients aged less than 18 years, patients with latent TB, cancer patients receiving chemotherapy treatment, pregnant and lactating women, patients transferred out who did not complete their treatment in HPP, patients who were diagnosed with TB than the diagnosis has been changed and patients with incomplete records were excluded (Figure 1).

Sample size calculation: A convenient sampling technique was adopted for the current study. All TB patients’ records registered at chest clinics (2914 records) from 2014–2018 were screened, and patients whose records complied with the inclusion and inclusion criteria (351 records) were included in the final analysis. 

### 2.2. Diagnosis and Treatment

Patients suspected of having TB were initially hospitalized to confirm or exclude the diagnosis. Once the patients’ diagnosis is confirmed, they have been discharged for treatment continuation unless they have medical complications that need monitoring. TB diagnosis was based on the guidelines of the Ministry of Health (MOH) Malaysia, which used the same procedure WHO raised [4,7]. 

The diagnosis of PTB and/or EPTB was based on the patient’s history reviewed by clinical expertise and clinical examination of the patient’s signs and symptoms, further supported by imaging (Chest X-ray) and laboratory tests. The diagnosis was confirmed by isolating Mycobacterium tuberculosis from clinical samples. Mycobacterial cultures were sent at the initiation of TB treatment to confirm the presence of Mycobacterium tuberculosis and to exclude drug-resistant TB. In the case of smear negative PTB, a chest X-ray is a sensitive tool used for identifying and excluding the diagnosis, as the clinical symptoms of smear negative PTB are similar to smear positive PTB. 

EPTB diagnosis depended on obtaining samples for Mycobacterium tuberculosis culture from the affected sites, such as cerebrospinal fluid (CSF), pleural fluid, fine needle aspiration (FNA), and/or biopsy from lymph nodes, pleura, and any other infected sites, to confirm the diagnosis and provide the drug susceptibility profile of the organism. 

Patients suspected of having MDR-TB should be initially evaluated for the presence of acid-fast bacilli (AFB) and rifampicin resistance using smear microscopy and Xpert Mycobacterium tuberculosis/rifampicin (MTB/RIF) assay. Upon positive sputum smear microscopy and rifampicin resistance, the patient should be enrolled in MDR-TB treatment with an empirical treatment regimen, and the specimen sample should be sent to a reference laboratory for a culture and drug sensitivity test (DST) against both first- and second-line anti-TB drugs (LJ Media and Automated/liquid media). Upon reception of DST results, patients should be switched from an empirical regimen to a standardized or DST-based individualized regimen. 

In Malaysia, TB treatment is fully under the umbrella of the national TB control program. Patients were treated with recommended standardized regimens following WHO TB guidelines. Patients diagnosed with PTB were treated with two months of intensive phase followed by 4 months for PTB and 4–12 months of maintenance phase for EPTB patients. The intensive phase regimen included isoniazid, rifampicin, pyrazinamide, and ethambutol (HRZE), whereas the maintenance phase regimen included isoniazid and rifampicin (HR). 

Treatment of MDR-TB depended either on a standard MDR-TB regimen (standardized approach) or an individually tailored regimen based on DST for second-line drugs. Intensive phase regimens consisted of 4 s-line anti-TB drugs that were proven to be more effective, including fluoroquinolones, aminoglycosides, ethionamide, and cycloserine with pyrazinamide. WHO recommended 8 months of the intensive phase for most patients. The anti-TB drugs used in treatment and their doses are shown in Table 1. 

### 2.3. Data Collection

Data of patients with confirmed active PTB and/or EPTB that were being followed up at the Chest Clinic were extracted manually from patients’ records in the chest clinic. A designed data sheet was used to collect patients’ sociodemographic and baseline clinical characteristics (age, gender, race, marital status, occupation, smoking, drinking, drug addiction, comorbidity, TB site, and TB history), pharmacotherapeutic options (receiving second-line anti-tuberculosis drugs, intensive and maintenance phase regimens, treatment duration, number of drugs taken, and patients’ compliance), microbiological (TB smear, TB culture, and sensitivity at baseline and Gene Xpert test), laboratory values, and drug-resistant patterns. 

Patients were given ID numbers (1–351) for coding, and then their data were entered into SPSS software as categorical and continuous variables for analysis. Based on WHO guidelines, patients’ treatment outcomes have been categorized into successful outcomes, including cured and treatment completed, and unsuccessful outcomes, including death, treatment failure, treatment failure, and default treatment [1]. Definitions of TB treatment outcomes are shown in Table 2. 

### 2.4. Statistical Analysis

To analyze the data, SPSS (version 23, IBM Corp., Armonk, NY, USA) was used. Descriptive statistics were conducted to analyze the data regarding the descriptive statistics for continuous data and categorical data. Continuous variables were presented as either mean ± standard deviation if they were normally distributed or median with their interquartile range (IQR) if not normally distributed using histogram and the Kolmogrov–Smirnov test. A variable is not normally distributed if “Sig.” < 0.05. Categorical variables are presented as percentages. The Cox proportional hazard regression model was used to determine the factors that are associated with time to mortality, having satisfied the assumptions of mutually exclusive events (dead and censored) and constant hazard ratios over time. Time (months) from TB diagnosis confirmed at hospital Pulau Pinang until death occurred, defined as time to event of interest. Additionally, Cox regression was used to determine the factors associated with MDR-TB development over time. Time to event of interest here is defined as time from TB diagnosis confirmed at hospital until MDR-TB diagnosis confirmed on the basis of drug sensitivity test results. Univariable Cox regression was performed by entering independent variables one by one to examine the association between these variables and MDR-TB development, as well as death, along with treatment duration. The entry of the independent variables into univariate analysis was based on the previously published studies, their possible relationship with the treatment outcomes, and recommendations from the clinical team and supervisors of the current study. Independent variables with a significant *p*-value (≤0.05) were included in a multivariable Cox regression analysis to see the independent risk factors of mortality and developing MDR-TB among TB patients. The Kaplan–Meier survival curve and log-rank test were performed to estimate the cumulative probability of survival of different variables. 

## 3. Results

### 3.1. Sociodemographic and Clinical Characteristics

At the study site, a total number of 351 TB patients received their treatment and had been followed up for treatment duration. The mean age of the patients was 48.84 ± 16.71 years. The majority of patients were males (74.4%), aged between 54–64 years (20.8%), Chinese (46.4%), married (64.4%), non-drinkers (86.0%), and non-drug addicts (83.2%). The majority of patients were registered as new cases (72.1%) and diagnosed with PTB (81.2%). Of the 285 PTB patients, 229 (65.2%) were smear positive upon initiation of treatment (Table 3).

### 3.2. Drug Resistant Patterns of TB Patients

The study participants were resistant to a median of one drug (range 1–4 drugs). At the baseline visit, the minority of participants were resistant to isoniazid (2.8%), rifampicin (4.0%), ethambutol (0.9%), pyrazinamide (0.9%), and streptomycin (1.7%) (Table 4).

### 3.3. Treatment Outcomes and Associated Factors of Mortality among TB Patients

Of 325 drug-susceptible TB patients, 245 (75.4%) achieved successful outcomes, 73 (22.5%) passed away, 6 (1.8%) defaulted, and one patient failed treatment. The median duration of drug-susceptible TB treatment was 6 months. The median time to death was 1 month (Table 5).

**Figure 2:**
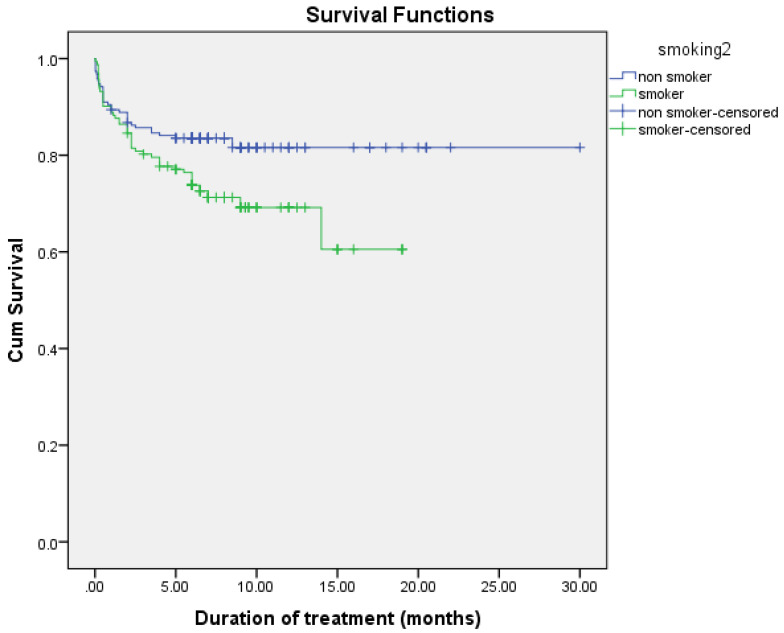
Cumulative probability of survival according to smoking.

**Figure 3:**
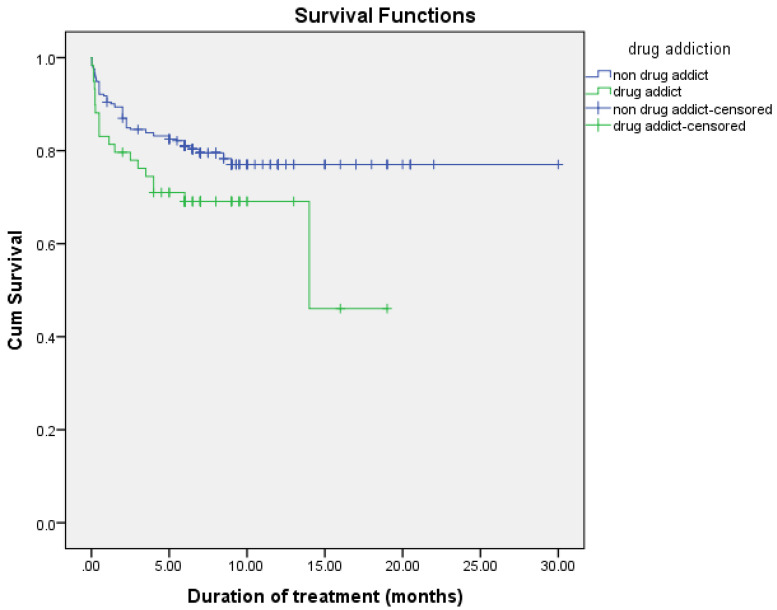
Cumulative probability of survival according to drug addiction.

**Figure 4:**
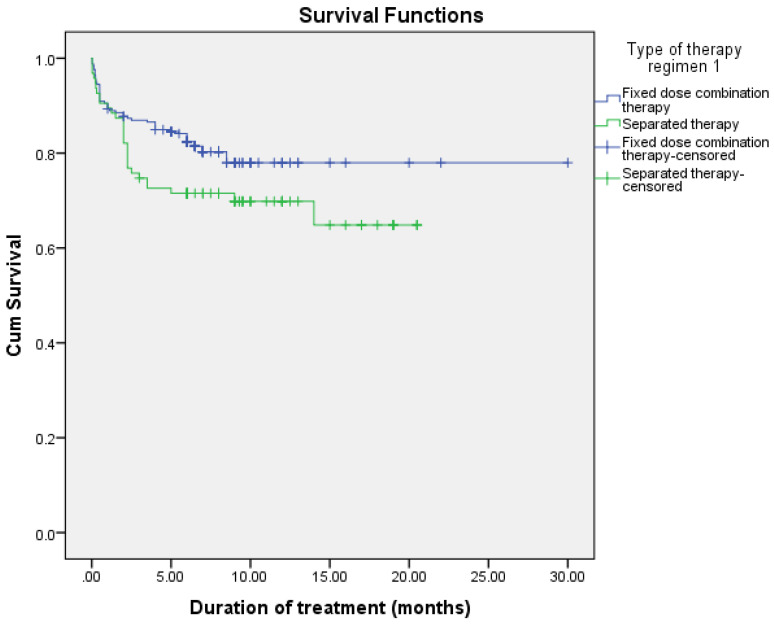
Cumulative probability of survival according to the intensive phase regimen.

**Figure 5:**
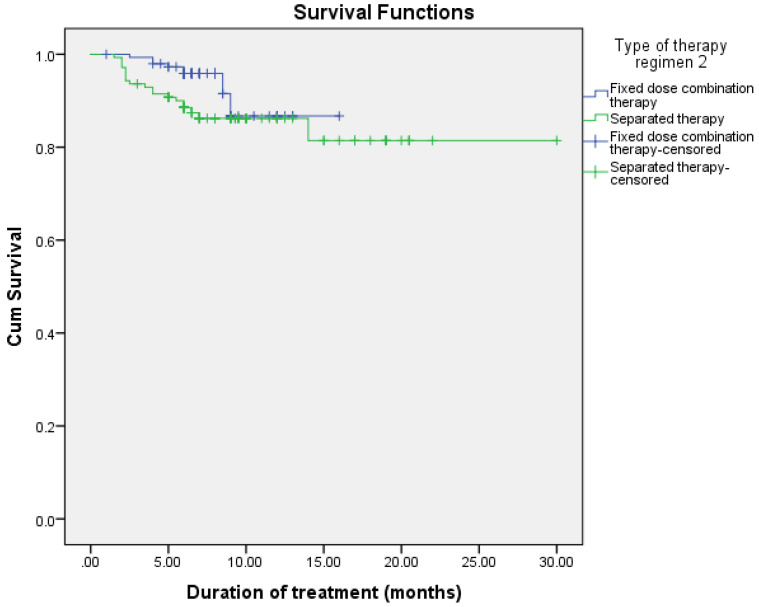
Cumulative probability of survival according to maintenance phase regimen.

The observed difference in survival experiences in different patient groups was assessed using the log-rank test. The results of the log-rank test show that the differences in the cumulative probability of survival of various factors, such as smoking, drug addiction, intensive phase regimen, and maintenance phase regimen, are statistically significant (*p* < 0.05), as shown in Figure 2, Figure 3, Figure 4 and Figure 5 and Table 6.

In univariable analysis, smoking (HR = 1.723, 95% CI = 1.081–2.746), drug addiction (HR = 1.652, 95% CI = 0.960–2.843), level of white blood cells at baseline (HR = 1.071, 95% CI = 1.026–1.118), neutrophils % (HR = 1.017, 95% CI = 1.006–1.029), urea (HR = 1.034, 95% CI = 1.020–1.048), and creatinine (HR = 1.001, 95% CI = 1.000–1.003) were significantly associated with increase risk of mortality. In addition, level of hemoglobin at baseline (HR = 0.809, 95% CI = 0.736–0.890), level of platelets (HR = 0.997, 95% CI = 0.996–0.999), lymphocytes % (HR = 0.943, 95% CI = 0.919–0.968), and albumin (HR = 0.957, 95% CI = 0.939–0.976) were significantly associated with decreased risk of mortality (Table 7).

In multivariable analysis, drug addiction (HR = 1.836, 95% CI = 1.019–3.309), levels of white blood cells at baseline (HR = 1.102, 95% CI = 1.057–1.148), and urea (HR = 1.029, 95% CI = 1.011–1.047) were independently associated with an increased risk of mortality. The level of platelets (HR = 0.996, 95% CI = 0.995–0.998) and albumin (HR= 0.964, 95% CI = 0.940–0.990) were associated with a decreased risk of mortality (Table 8).

### 3.4. Treatment Outcomes and Associated Factors for Developing Multidrug Resistant TB

Among the 351 patients included in the final analysis, 26 (7.4%) were diagnosed with MDR-TB. Of the 21 (80.8%) patients who achieved successful outcomes, 5 (19.2%) passed away. The median duration of treatment was 12 months (range 2–20 months) (Table 5).

In univariable analysis, the variables that had a statistically significant association with increased risk of MDR-TB were being single (HR = 0.457, 95% CI = 1.960–10.548), alcohol consumption (HR = 7.452, 95% CI = 3.206–17.324), and relapsed TB cases (HR = 3.035, 95% CI = 1.306–10.196). In addition, smoking (HR = 0.088, 95% CI = 0.025–0.301), not being a prisoner (HR = 0.270, 95% CI = 0.075–0.978), not having a history of TB (HR = 0.400, 95% CI = 0.168–0.952), no growth of MTB (HR = 0.300, 95% CI = 0.093–0.971) and receiving an anti-TB regimen as separated therapy (HR = 0.101, 95% CI = 0.029–0.355) were associated with decrease risk of MDR-TB development (Table 9).

In multivariable analysis, the variables that emerged as independent risk factors associated with the development of MDR-TB were drinking (HR = 7.591, 95% CI = 3.097–18.610), relapsed cases (HR = 3.035, 95% CI = 1.028–8.957) and being single (HR = 6.817, 95% CI = 2.599–17.879) (Table 10).

## 4. Discussion

Our research looked into the factors that contribute to mortality among TB patients, as well as the factors associated with MDR-TB development. Since then, tuberculosis treatment has been problematic and difficult to apply properly because of long duration and compliance issues due to anti-tubercular drugs’ side effects. Some of the side effects are self-limiting, but others necessitate treatment discontinuation. Although rare, thrombocytopenia is one of the most common and serious life-threatening side effects. Thrombocytopenia is caused by the formation of antibodies that bind to platelets and initiate the complement cascade, causing these cells to lyse. These antibodies also suppress the production of platelets [8]. This study demonstrated that thrombocytopenia significantly increased the risk of mortality among TB patients. [9] reported a low platelet count as a significant risk factor for mortality.

Mortality among patients suffering from any infection has been linked to WBC count [10]. As a result, elevated WBC counts in TB patients may be related to the severity of lung involvement and provide an estimated risk of mortality [11]. During TB infection, the body produces leukocytes and macrophages as part of the immune mechanism to defend the body against invading *Mycobacterium tuberculosis*, which raises the total number of WBC [12]. The current study findings documented that every 10^3^ cells/µL increment in WBC above the normal range increased the risk of death by 1.102. In line with our results, a study conducted in Malaysia pointed out that the risk of death increased by 1.12 times for every 10^3^ cells per microliter unit increase in total WBC [13]. Other studies have indicated that WBC levels are a significant risk factor correlated with TB mortality [11,14]. Pneumonia ranked the second cause of death among the study population. The WBC count elevation has been reported as a predictor of the severity of pneumonia, one of the leading causes of death in TB patients [11].

The present findings indicated that the level of urea was a strong significant predictor of death. Increased levels of urea were found to be significantly associated with mortality [11]. We can relate elevated urea levels to kidney dysfunction, where renal clearance is reduced due to renal impairment/failure or chronic kidney disease (CKD). Urea levels may also increase in other conditions, such as upper GI bleeding [15]. As the prevalence of CKD in our cohort was 5.69%, where 20 patients developed CKD while receiving anti-tuberculosis treatment, further research is needed to determine the relationship between CKD and TB. Strategies for the prevention, detection, and treatment of TB-CKD cannot be refined without advanced understanding.

Hypoalbuminemia showed a statistically significant relationship with death; patients who had hypoalbuminemia were more likely to pass away than patients who did not. Matching our results, lower albumin levels showed an association with non-survivors (*p*< 0.001) compared to survivors. Low levels of albumin were found to be significantly correlated with the hazard of death [9,16]. Furthermore, hypoalbuminemia was considered a predictor of mortality in community-acquired pneumonia (CAP), which is not surprising considering that albumin is involved in inflammation and indicates malnutrition [17]. The initial value of serum albumin might also be a result of malnutrition or an underlying disease that can worsen the nutritional status of the patient [18]. As a result of malnutrition, deceased patients’ immune systems were suppressed and were therefore sicker upon initial presentation.

As for drug addiction characteristics, the findings of this study revealed that patients who had a history of addiction or were current addicts had a 1.895 times higher risk of death than those who had never abused drugs. In line with our findings, deceased patients were more likely to abuse heroin IV than living patients, and addiction was correlated with all causes of death in tuberculosis patients [19,20]. Supporting our results, a history of addiction was a statistically significant risk factor for death within the first year after diagnosis in Hong Kong, Russia, and Iran [21,22,23]. Poor adherence of drug users, default treatment, and poor access to health services can create challenges for the treatment of the drug addict population [24]. As drug users have a higher likelihood of default treatment than non-drug users, the increased hazard of death can be attributed to the increased prevalence of alcohol misuse, chronic viral hepatitis, and anti-tubercular drug-induced hepatotoxicity that injectable drug users exhibit [25,26].

Drug resistance TB has emerged as a serious health issue undermining global efforts to control TB and reduce the spread of the disease, in addition to making TB treatment more complicated. Thus, we aimed to study the risk factors associated with the development of MDR-TB in TB patients. In the current study, relapsed TB cases were more likely to develop MDR-TB than newly registered cases that had never had exposure to anti-TB treatment. In agreement with our findings, a history of treatment with anti-tubercular drugs has been mentioned in the previous literature as a risk factor for MDR-TB development in common with other risk factors [27,28,29,30]. Relapsed TB cases were found to be more likely to achieve unsuccessful treatment outcomes [31,32]. As the history of being treated with anti-TB drugs raises the hazard of resistance to many drugs, more attention must be paid to identifying the underlying causes of treatment failure and default treatment, which may decrease the incidence of retreatment among tuberculosis patients [28]. The link between prior anti-TB exposure and the emergence of drug resistance could be due to the fact that prior anti-TB exposure suppresses only the growth of susceptible bacilli. On the other hand, it may allow for the multiplication of pre-existing drug-resistant mutants [33].

One of the sociodemographic characteristics related to tuberculosis is marital status. Our findings reported that single patients were at 6.817 times higher risk of developing MDR-TB than married patients, which was in agreement with other studies that revealed that single individuals were at higher risk of developing MDR-TB than married individuals [34,35]. Even though there is no biological relationship between the marital status of the patients and TB, when compared to married people, single people are more likely to be infected with tuberculosis or MDR-TB strains. Being at high risk can still be due to a lack of social support or participation in high-risk behaviors, such as alcohol and drug addiction [36].

Aside from the previously mentioned risk factors, alcohol consumption has been significantly associated with developing MDR-TB. Patients who had a history of drinking or were current drinkers had a 7.591 times higher risk of MDR-TB than those who had never drank. Drinkers had a higher risk of MDR-TB, according to the results of a study conducted in China and Uganda [37,38]. Alcohol use was a contributing factor in TB treatment interruption, which is the main factor in MDR-TB development [39]. Many explanations could stand behind the impact of alcohol consumption on MDR-TB. Poorer treatment outcomes, including default treatment, mortality, and treatment failure, have been observed in TB patients who have consumed alcohol and MDR-TB, while treatment failure has been identified as a significant risk factor for drug resistance [38]. In addition, the consequences of alcohol ingestion can result in liver damage, weakened immunity, and nutritional deficiency, contributing to sensitive and resistant TB infection [40,41,42].

The findings of the current study highlighted patients’ groups that are at high risk of achieving unsuccessful TB treatment outcomes. As MDR-TB has proven to be a major influencer on patients’ health and safety, more attention must be paid to the factors leading to relapse and overcoming them in order to minimize the risk of MDR-TB occurrence in TB patients. A must improve public awareness about the worsening effect of alcohol on TB treatment and the importance of psychological support from the patient’s family to adhere to TB treatment. Moreover, the government should implement more restricted rules to minimize the availability of drugs in the population hands and to increase the awareness of people regarding the hazard of drugs. Additionally, malnutrition status was a risk factor for death among TB patients, so monthly counseling and material support in the form of food is suggested.

The study has many limitations that must be recognized. This study targeted only patients registered with HPP, which may not cover the true proportion of the Malaysian population. Being a unicentral study and a convenient sample technique limits the generalization of the results to the entire Malaysian population. An unequal proportionality among several variables, including age, ethnicity, and gender, might influence the final results in comparison with randomized control trials (selection bias). Due to the retrospective nature of the present study, it was impossible to record certain sociodemographic characteristics of the patients who passed away and default treatment (information bias), which might enhance the findings of the study. It was difficult to set a control group that needed further clinical trials to support the study results. The aforementioned limitations might affect the extent to which the current findings are accurate and consistent and can be reproduced under the same methodology.

## 5. Conclusions

Eventually, patients with a history of addiction or who were currently addicted had a higher mortality rate than non-addict patients. Among TB patients, drug addiction, white blood cell count, urea levels, platelet counts, and albumin levels were all risk factors for death. Multidrug-resistant tuberculosis developed in 26 (6.0%) out of 351 patients. MDR-TB development was significantly related to relapsed cases, alcohol consumption, and being single.

## Figures and Tables

**Table 1:** Anti-TB drugs and dose ranges for TB patients.

Drug	Daily Dose (mg/day)
^1^ Akurit 2	2–5 tab/day
^2^ Akurit 4	2–5 tab/day
Pyridoxine	10–50
Isoniazid	150–300
Rifampicin	300–600
Ethambutol	400–1600
Pyrazinamide	250–2000
Streptomycin	500–1000
Levofloxacin	250–1000
Ethionamide	250–500
Kanamycin	500–1000
Moxifloxacin	400–800
Clofazimine	100–200
Cycloserine	250
Meropenem	1000
Augmentin	1200
Bedaquiline	200
Linezolid	600
Ofloxacin	40
Clarithromycin	500

^1^ Akurit 4: H 75 mg, R 150 mg, Z 400 mg, E 275 mg, ^2^ Akurit 2: H 75 mg, R 150 mg.

**Table 2:** Classification of tuberculosis treatment outcomes [1].

Outcome	Definition
Cured	A pulmonary TB patient with bacteriologically confirmed TB at the beginning of treatment who was smear- or culture-negative in the last month of treatment and on at least one previous occasion.
Treatment completed	A TB patient who completed treatment without evidence of failure BUT with no record to show that sputum smear or culture results in the last month of treatment and on at least one previous occasion were negative, either because tests were not done or because results are unavailable.
Died	A TB patient who dies for any reason before starting or during the course of treatment.
Treatment failed	A TB patient whose sputum smear or culture is positive at month 5 or later during treatment.
Lost to follow up	A TB patient who did not start treatment or whose treatment was interrupted for 2 consecutive months or more.
Not evaluated	A TB patient for whom no treatment outcome is assigned. This includes cases “transferred out” to another treatment unit, as well as cases for whom the treatment outcome is unknown to the reporting unit.
Treatment success	The sum of cured and treatment completed.

**Table 3:** Sociodemographic and clinical characteristics of tuberculosis patients (N = 351).

Variable		Median (IQR)	No. (%)
Gender	Female	-	90 (25.6)
	Male		261 (74.4)
Age (years)	18–24	51.0 (74.0)	56 (15.9)
	>24–34		48 (13.7)
	>34–44		54 (15.4)
	>44–54		60 (17.1)
	>54–64		73 (20.8)
	≥64		60 (17.1)
Race	Malay	-	126 (35.9)
	Chinese		163 (46.4)
	Indian		45 (12.8)
	* Other		17 (4.8)
Marital status	Married	-	226 (64.4)
	Single		125 (35.6)
Smoking Habbit	Non-smokers	-	189 (53.8)
	Smokers		162 (46.2)
Alcohol consumption	No	-	302 (86.0)
	Yes		49 (14.0)
Drug addiction	No	-	292 (83.2)
	Yes		59 (16.8)
Imprisoned	No	-	335 (95.4)
	Yes		16 (4.6)
Diabetes mellitus	Non-diabetics	-	260 (74.1)
	Diabetics		91 (25.9)
Human immunodeficiency virus	Non-reactive	-	310 (88.3)
	Reactive		41 (11.7)
Hepatitis B virus	Non-reactive	-	338 (96.3)
	Reactive		13 (3.7)
Hepatitis C virus	Non-reactive	-	323 (92.0)
	Reactive		28 (8.0)
Case category	New	-	253 (72.1)
	Relapse		98 (27.9)
History of TB	No history	-	256 (72.9)
	History		95 (27.1)

* Other: Indian, Chinees, Indonesia, Myanmar, Philbin.

**Table 4:** Drug resistance patterns of tuberculosis patients (N = 351).

Variable		No. (%)
DST for isoniazid	INH susceptible	100 (28.5)
	INH resistant	10 (2.8)
	Not applicable	241 (68.7)
DST for rifampicin	RIF susceptible	96 (27.4)
	RIF resistant	14 (4.0)
	Not applicable	241 (68.7)
DST for ethambutol	ETH susceptible	105 (29.9)
	ETH resistant	3 (0.9)
	Not applicable	243 (69.2)
DST for streptomycin	STM susceptible	103 (29.3)
	STM resistant	6 (1.7)
	Not applicable	242 (68.9)
DST for pyrazinamide	PZA susceptible	6 (1.7)
	PZA resistant	3 (0.9)
	Not applicable	342 (97.4)

**Table 5:** Treatment outcomes of study participants as per WHO guidelines (N = 351).

Outcomes	Drug-Susceptible TB (N = 325)	MDR-TB (N = 26)
Death	73 (22.5%)	5 (19.2%)
Cure	171 (52.6%)	-
Treatment completed	74 (22.8)	21 (80.8%)
Treatment failure	1 (0.3%)	-
Loss of follow up/default treatment	6 (1.8%)	-

**Table 6:** The log-rank test of factors influencing the survival of TB patients (*n* = 351).

Variable		Chi-Square	df	Sig. (Log-Rank)
Gender	Female Male	2.050	1	0.152
Smoking habit	Non-smokers Smokers	5.743	1	**0.017**
Alcohol consumption	Non-drinkers Drinkers	0.404	1	0.525
Drug addiction	Non-drug addicts Drug addicts	4.450	1	**0.035**
History of TB	History No history	0.093	1	0.760
Diabetes mellitus	Diabetics Non-diabetics	1.365	1	0.243
Intensive phase regimen	Fixed-dose combination Separated therapy	3.892	1	**0.049**
Maintenance phase regimen	Fixed-dose combination Separated therapy	3.834	1	**0.000**

**Table 7:** Univariable Cox regression analysis of risk factors for mortality among TB patients (N = 351).

Variable		Treatment Outcome		Hazard Ratio (95% CI)	*p*-Value
		Alive, N (%)	Died, N (%)		
Gender	Female Male	70 (27.8) 182 (72.2)	15 (20.5) 58 (79.5)	Referent 1.511 (0.844–2.705)	0.165
Smoking habit	Non-smokers Smokers	151 (59.9) 101 (40.1)	32 (43.8) 41 (56.2)	Referent 1.723 (1.081–2.746)	**0.022**
Alcohol consumption	Non-drinkers Drinkers	224 (88.9) 28 (11.1)	65 (89.0) 8 (11.0)	Referent 1.289 (0.708–2.349)	0.407
Drug addiction	Non-drug addicts Drug addicts	216 (85.7) 36 (14.3)	54 (74.0) 19 (26.0)	Referent 1.652 (0.960–2.843)	**0.050**
History of TB	History No history	54 (21.4) 198 (78.6)	23 (31.5) 50 (68.5)	Referent 0.960 (0.576–1.599)	0.875
Diabetes mellitus	Diabetics Non-diabetics	68 (27.0) 184 (73.0)	17 (23.3) 56 (76.7)	Referent 1.363 (0.783–2.373)	0.274
Intensive phase regimen	Fixed-dose combination Separated therapy	204 (81.0) 48 (19.0)	47 (66.2) 24 (33.8)	Referent 1.571 (0.976–2.5280)	**0.063**
Maintenance phase regimen	Fixed-dose combination Separated therapy	141 (56.0) 102 (40.5)	8 (11.0) 15 (20.5)	Referent 2.130 (0.914–4.963)	0.080
White blood cells, median (IQR)		8.7 (30.6)		1.071 (1.026–1.118)	**0.002**
Haemoglobin		11.9 (13.1)		0.809 (0.736–0.890)	**0.000**
Platelets		309.0 (977.0)		0.997 (0.996–0.999)	**0.000**
Neutrophils %		71.5 (132.9)		1.017 (1.006–1.029)	**0.003**
Lymphocytes %		17.3 (80.7)		0.943 (0.919–0.968)	**0.000**
Albumin		28.0 (34.3)		0.957 (0.939–0.976)	**0.000**
ALT		18.0 (796.0)		1.002 (0.999–1.006)	0.157
Urea		3.9 (78.7)		1.034 (1.020–1.048)	**0.000**
Creatinine		72.0 (1322)		1.001 (1.000–1.003)	**0.008**

CI, confidence interval; TB, tuberculosis; ALT, alanine aminotransferase.

**Table 8:** Multivariable Cox regression analysis of risk factors for mortality among tuberculosis patients.

Variable	HR (95% CI)	*p*-Value
Drug addiction	1.836 (1.019–3.309)	0.034
White blood cells	1.102 (1.057–1.148)	0.000
Platelets	0.996 (0.995–0.998)	0.000
Albumin	0.964 (0.940–0.990)	0.006
Urea	1.029 (1.011–1.047)	0.002

**Table 9:** Univariable Cox regression analysis of risk factors for multidrug resistance among TB patients (N= 351).

Variable		Multi-Drug Resistant TB		Hazard Ratio (95% CI)	*p*-Value
		Yes (N = 26)	No		
Gender	Female Male	5 (19.2) 21 (80.8)	85 (26.2) 240 (73.8)	Referent 2.024 (0.743–5.512)	0.168
Race	Malay Non-Malay	4 (15.4) 22 (84.6)	121 (37.2) 204 (62.8)	Referent 2.067 (0.692–6.174)	0.193
Marital status	Married Single	9 (34.6) 17 (65.4)	217 (66.8) 108 (33.2)	Referent 4.547 (1.960–10.548)	**0.000**
Smoking	Non-smokers Smokers	6 (23.1) 20 (76.9)	183 (56.3) 142 (43.7)	Referent 0.088 (0.026–0.301)	**0.000**
Drinking	Non-drinkers Drinkers	13 (50.0) 13 (50.0)	289 (88.9) 36 (11.1)	Referent 7.452 (3.206–17.324)	**0.000**
Drug addiction	Non-drug addicts Drug addicts	22 (84.6) 4 (15.4)	270 (83.1) 55 (16.9)	Referent 1.454 (0.491–4.305)	0.499
Imprisoned	Prisoners Non-Prisoners	3 (11.5) 23 (88.5)	13 (4.0) 312 (96.0)	Referent 0.270 (0.075–0.978)	**0.046**
History of TB	History No history	18 (69.2) 8 (30.8)	77 (23.7) 248 (76.3)	Referent 0.400 (0.168–0.952)	**0.038**
Case category	New Relapse	5 (19.2) 21 (80.8)	248 (76.3) 77 (23.7)	Referent 3.649 (1.306–10.196)	**0.014**
MTB culture at baseline	Growth No growth	6 (5.0) 6 (50.0)	8 (12.3) 57 (87.7)	Referent 0.300 (0.093–0.971)	**0.044**
Intensive phase regimen	Fixed-dose combination Separated therapy	3 (12.0) 22 (88.0)	269 (82.8) 56 (17.2)	Referent 0.101 (0.029–0.355)	**0.000**

MTB, Mycobacterium tuberculosis.

**Table 10:** Multivariable Cox regression analysis of risk factors for multidrug resistance among TB patients.

Variable	HR (95% CI)	*p*-Value
Relapse TB	3.035 (1.028–8.957)	0.044
Alcohol consumption	7.591 (3.097–18.610)	0.000
Being single	6.817 (2.599–17.879)	0.000

## Data Availability

Not applicable.
